# Cutaneous Basal Cell Carcinoma with Lymph Node and Pulmonary Metastases

**DOI:** 10.1155/2018/3485326

**Published:** 2018-04-24

**Authors:** Renate U. Wahl, Claudio Cacchi, Albert Rübben

**Affiliations:** ^1^Department of Dermatology, Euregional Skin Cancer Center, University Hospital of the RWTH-Aachen, Aachen, Germany; ^2^Department of Pathology, University Hospital of the RWTH-Aachen, Aachen, Germany

## Abstract

Basal cell carcinoma (BCC) is the most common skin cancer. Metastatic BCC is an extraordinary rare finding observed in only 0.5% of all cases. Until the introduction of the small molecule hedgehog inhibitor vismodegib, patients with metastatic BCC were treated with chemotherapy, most frequently platinum-based with mixed responses to therapy. We present the case of a 55-year-old Caucasian man who suffered from BCC on his left arm with lymph node and pulmonary metastases. Sonic hedgehog blockade with vismodegib only induced a short remission, and the patient succumbed to the cancer.

## 1. Introduction

Basal cell carcinoma (BCC) of the skin is the most common human cancer in Caucasians. Its high incidence can be explained by genetics as inactivation of only one-signal transduction pathway; that is, the sonic hedgehog (SHH) signaling, either by one activating mutation in the *SMO gene* or by two inactivating mutational events targeting the *PTCH1 gene*, seems to be sufficient for cancer formation. Consequently, blockade of SHH signaling has been developed as systemic treatment of advanced BCC [1]. Although BCC can induce extensive and lethal local tissue destruction, formation of metastases is exceedingly rare. Metastasis frequency is estimated to range from 0.003% to 0.5% [2]. Low frequency of metastases of BCC stands in contrast to the very high mutation load demonstrated in sporadic BCC and to a lesser extent also in syndromic BCC [3–5]. On the other hand, most mutations demonstrate an UV-signature and one might speculate that a cancer driven mostly by exogenous UV-induced mutations might be less prone to acquire the additional mutations necessary for the metastatic process than a cancer evolving primarily through a mutator phenotype or through marked aneuploidy. Nevertheless, genetic instability in BCC is not excluded, and mutations of the caretaker gene P53 and chromosomal instability have been observed frequently in BCC [3].

We would like to present an additional case of metastatic and fatal BCC in a surprisingly young male patient.

## 2. Case Presentation

In January 2015, a 55-year-old Caucasian man was admitted to our department with a 12-year history of a slow-growing ulcerating tumor of the left forearm. The patient had no comorbidities and no prior skin cancer history but a 40-year history of cigarette smoking. The only skin cancer risk factor was a Fitzpatrick skin type II. The patient had avoided medical care due to diffuse fear and distrust in the health care system, but within the last couple of months, the patient developed an ulceration of the left axilla which ultimately forced him to seek medical advice.

**Figure 1:**
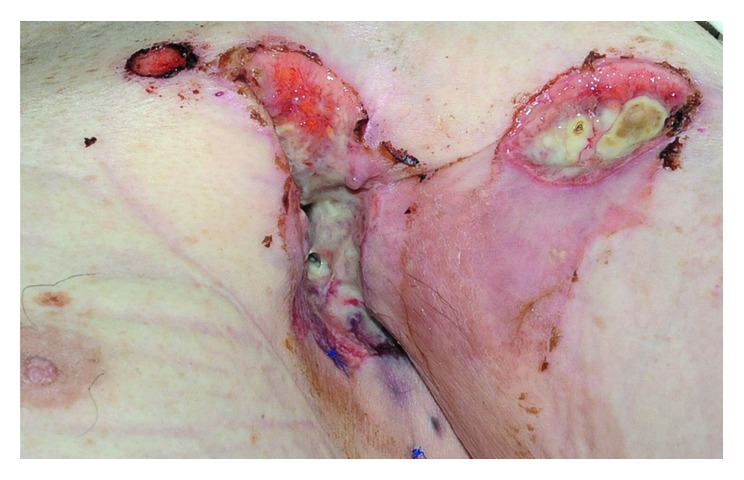
Axillary ulcerating lymph node metastases of cutaneous basal cell carcinoma located on the left forearm.

**Figure 2:**
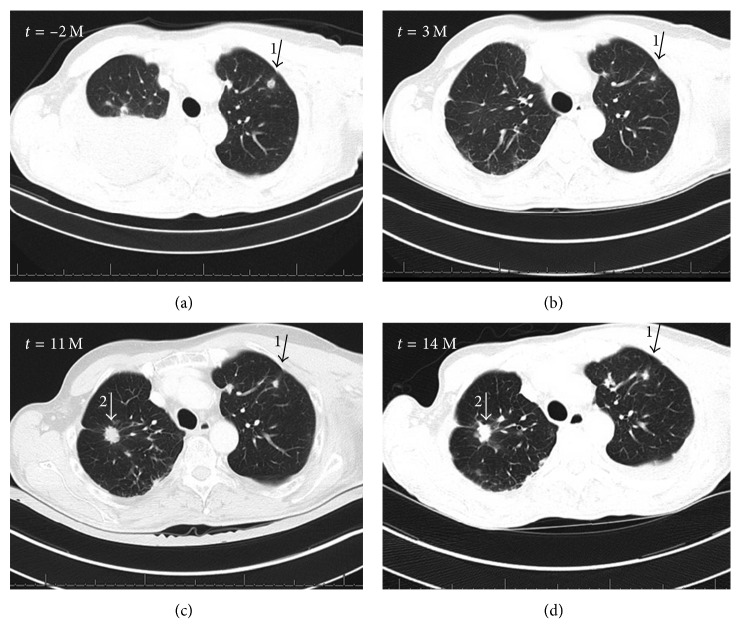
Computer tomography demonstrating pulmonary metastases two months before treatment (*t* = −2 M), with partial regression after 3 months of vismodegib treatment (*t* = 3 M, arrow 1) and with progressive disease after 11 and 14 months (arrow 1). Arrow 2 indicates new metastasis first visible on the CT scan after 11 months of treatment.

**Figure 3:**
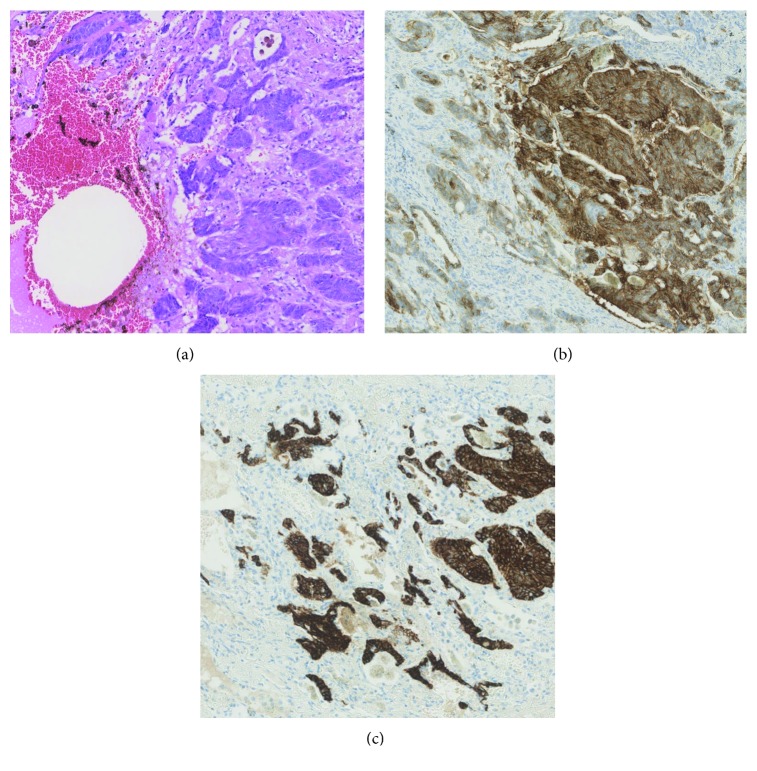
(a) H&E staining of pulmonary metastatic tissue demonstrating typical hyperchromatic basaloid cells (×200). (b) Positive BerEP4 staining of tumor tissue (×200). (c) Positive CK 5/6 staining of tumor tissue (×200).

On initial presentation, we observed an 8 × 15 cm measuring wound on the left forearm and a 5 × 8 cm measuring and 3-4 cm deep ulceration of the left axilla (Figure 1). Several dark brown papules were seen on the left thoracic wall. We performed biopsies from the lesion on the left forearm, the axillary ulcers, and the papules on the thoracic wall with the histopathological finding of a basal cell carcinoma (BCC) in all lesions. A metastasized BCC was diagnosed, and subsequent radiographic diagnostics revealed a pleural effusion in the right lung and multiple suspicious lesions in both lungs (Figure 2). Lung metastases of the BCC were confirmed by bronchoscopy and pleural biopsy. The carcinomatous pulmonary tissue matched to the lesion on the patient's left forearm by histology and immunohistochemistry with strong marking of CK 5/6 and a positive reaction to BerEP4 (Figure 3).

An oral medication with the SHH pathway inhibitor vismodegib, 150 mg/day, was started, and the patient's health state improved temporarily. The patient suffered from therapy-induced alopecia as well as from mild muscle spasms and mild dysgeusia which were first reported after 5 months of treatment, but vismodegib dosage does not need to be reduced. Initially, the cutaneous and pulmonary metastases decreased in size (Figure 2, arrow 1), but 6 months after starting vismodegib, radiotherapy of refractory and slowly progressive ulcerations of the left axilla and the forearm had to be performed. Pulmonary metastases demonstrated progression 9 months after starting vismodegib (Figure 2). In order to rule out a secondary pulmonary neoplasm, an additional metastatic pulmonary lesion was excised and histopathology again confirmed metastatic BCC.

Therapy with vismodegib was discontinued 11 months after initiation of treatment in order to switch to chemotherapy. However, the patient soon developed pneumonia and died of septic infection 4 months after vismodegib discontinuation and before chemotherapy could be initiated.

## 3. Discussion

Basal cell carcinoma (BCC) is the most commonly diagnosed skin cancer, but metastatic BCC is an extraordinary rare finding. Metastatic BCC was first described by Beadles in 1894 [6]. In 1951, Lattes and Kessler provided narrow criteria for the definition of true metastatic BCC cases [7]. Less than 400 cases of true metastatic BCCs have been described till now [2].

Metastatic BCC spreads into lymph nodes by hematogenous dissemination, with the lung being the most often affected organ [2, 8, 9]. Metastatic BCC has a poor prognosis with mean survival rates of 3 years in cases of locoregional lymphatic metastases and of 8 months in patients with distant metastases. Large primary tumors, invasion of blood vessels or of perineural spaces, location in the head and neck region, multiple recurring or primary tumors, condition after radiotherapy, immunosuppression, and fair skin as well as male gender have been described as risk factors for developing a metastatic BCC [2, 9].

Immunohistochemistry should be performed additionally to routine histopathology in order to differentiate basal cell carcinoma from squamous cell carcinoma (SCC) of the skin or from basosquamous carcinoma, which presents a mixture of squamous and basaloid components. In our case, a strong reaction to CK 5/6 and a weaker reaction to BerEP4 were found. Cytokeratin 5/6 antibodies frequently show immunoreactivity in BCC as well as in squamous cell carcinoma of the skin [10]. However, pulmonary adenocarcinomas and pulmonary small cell cancers do not express CK 5/6, thereby allowing differentiation from primary pulmonary carcinoma [10]. BerEP4 is an antibody to the transmembrane epithelial adhesion molecule EpCAM. EpCAM is present on all nonsquamous epithelial cells [11]. Accordingly, BerEP4 is a sensitive marker of BCC but does not stain cutaneous SCC [12]. Clinics as well as the BerEP4 staining in our patient's cutaneous and pulmonary biopsies supported the diagnosis of a metastatic BCC.

Therapeutic standard for localized BCC is micrographic controlled surgery. Topical treatment with 5-FU or imiquimod, photodynamic therapy, cryotherapy, or laser therapy can be considered for superficial BCC. In cases of inoperable BCCs due to size, location, age, or medical history of the patient, radiotherapy is a palliative option. Until January 2012, no specific systemic therapy was available for patients with locally advanced or metastatic BCC. Patients were treated with chemotherapy, most frequently platinum-based or with more than one chemotherapy agent with mixed responses [1, 2, 8]. In the US, vismodegib was introduced as the first targeted therapy for BCC on January 30, 2012. Locally advanced BCCs showed a complete response to vismodegib in 21% and an assessed response rate of 43%, whereas the assessed response rate in patients with metastatic BCC was only 30% [13]. Muscle spasm, alopecia, dysgeusia, weight loss, and fatigue are the most common adverse side effects of vismodegib. Similar to other targeted therapeutic agents, development of resistance to vismodegib can be observed during treatment or after therapy-free periods. The mean response duration is 7.6 months [13]. Unfortunately, with loss of efficacy, the disease progresses rapidly causing the patients' death in short term as mirrored by our case report.

Activation of the RAS-MAPK pathway has been identified as one mechanism of resistance to SSH blockade [14] which is in line with the observation that some BCC have responded to EGFR blockade of the RAS-MAPK pathway [15]. Ongoing studies are exploring the immunologic approach by PDL1 blockade in advanced BCC, and preliminary results have demonstrated some efficacy in metastatic BCC [16]. A strong medical need persists to develop effective treatments for advanced and metastatic cutaneous basal cell carcinoma.
